# Evaluation of local and systemic side effects of Turkovac vaccine in adults

**DOI:** 10.55730/1300-0144.5657

**Published:** 2023-05-03

**Authors:** Bülent Nuri KALAYCI, Doğu KARAHAN

**Affiliations:** 1 Department of Dermatology, Faculty of Medicine, Malatya Turgut Özal University, Malatya, Turkiye; 2 Department of Internal Medicine, Faculty of Medicine, Malatya Turgut Özal University, Malatya, Turkiye

**Keywords:** Side effects, ERU-CoV, Turkovac, vaccine, COVID-19

## Abstract

**Background/aim:**

COVID-19 is a respiratory disease that caused a pandemic after reportedly emerging from Wuhan, China, in December 2019. Different types of COVID-19 vaccines such as viral vectors, mRNA, and inactivated vaccines have been produced since the beginning of the pandemic. Turkovac is an inactive COVID-19 vaccine developed and produced in Türkiye. We conducted our study to investigate the local and systemic side effects of the Turkovac vaccine.

**Materials and methods:**

A cross-sectional survey-based study was conducted to collect data on the postvaccine side effects in people aged over 18 who were vaccinated with Turkovac, between March and June 2022, in the Malatya Turgut Özal University Research and Training Hospital. A 54 question, multiple-choice questionnaire was used to collect demographic data from the participants and identify the possible local and systemic side effects after Turkovac vaccine administration.

**Results:**

Of the 403 participants included in the study, 134 (33.3%) were female and 269 (66.7%) were male with a mean age of 47.7 ± 13.7. The most common local side effects observed after vaccination were pain at the injection site (22.8%) and local swelling and redness (0.5%). Systemic side effects included weakness or fatigue (4.2%), muscle or joint pain (2%), headache (1.7%), fever (1%), cough (0.25%), lymphadenopathy (0.25%), and urticaria (0.25%). Side effects were most commonly observed within the first 24 h. We found that for participants under 47 years of age, female sex, chronic diseases, and regular medication use were associated with the risk of suffering side effects.

**Conclusion:**

Our study revealed that Turkovac is a generally well-tolerated vaccine and had no side effects. More studies are required to evaluate Turkovac’s side effects in other populations.

## 1. Introduction

COVID-19 is a systemic disease caused by severe acute respiratory syndrome-coronavirus-2 (SARS-CoV-2) that reportedly first appeared in Wuhan, China, in December 2019, causing a global pandemic [1]. According to estimates, 609,717,922 cases and 6,502,052 deaths have been reported worldwide up until September 3, 2022^1^. As COVID-19 can spread rapidly through contact with respiratory droplets from an infected person, the World Health Organization (WHO) recommends maintaining a physical distance of at least one m, wearing a facemask, and washing hands regularly with soap and water to control the spread of infection^2^. Vaccination is the most effective method to protect against COVID-19. Various types of COVID-19 vaccines have been developed since the beginning of the pandemic including viral vectors, mRNA, and inactivated vaccines. Inactivated vaccines are produced by the propagation of SARS-CoV-2 in cell culture followed by inactivation of the virus [2]. The first inactivated SARS-CoV-2 vaccine, CoronaVac (Sinovac Life Sciences, Beijing, China), was initially used in vaccine trials in April 2020, and was subsequently included in the vaccination programme in Türkiye after receiving emergency approval^3^.

Turkovac (ERU-CoV, Kayseri, Türkiye) is an inactivated SAR-CoV-2 vaccine developed with the support of the Turkish Health Institutes (TÜSEB), also produced in Türkiye [3]. ERU-CoV-Vac is formulated in aluminium hydroxide and produced using Vero E6 cell cultures, was renamed to Turkovac upon the start of phase 3 studies [4]. The Turkovac vaccine was granted emergency use approval in December 2021, by the Ministry of Health, Turkish Medicines and Medical Devices Agency (TITCK), and it was included in the vaccination programme^4^. Although the efficacy of Turkovac has been proven in animal experiments and clinical studies, the literature remains limited [4,5]. We propose a study to investigate possible local and systemic side effects that might occur after Turkovac vaccine administration.

## 2. Materials and methods

The study consisted of 403 individuals over the age of 18 who were vaccinated with Turkovac at Malatya Turgut Özal University Faculty of Medicine Training and Research Hospital between March and June 2022. The Ministry of Health Scientific Research Platform (Bülent Nuri Kalaycı-2022-02-22T18_00_28) approved the study and we obtained permission from the local ethics committee (protocol number: 2022/14, date: 28.04.2022). Our study was carried out in accordance with the Declaration of Helsinki. Individuals who made vaccination appointments via the Central Hospital Appointment System were contacted by phone in the order of their application.

Power analysis accepting an effect size of 0.5, a power of 95%, and a margin error of 5% determined a sample size of 176 individuals. Individuals were enrolled after at least 3 weeks had passed post-Turkovac vaccination and informed consent was obtained from all participants. A multiple-choice questionnaire of 54 questions was completed and participants had to show capacity to answer the survey questions competently to be included in the study. The first part of the questionnaire queried sex, age, occupation, additional systemic diseases, allergies to medication, vaccines, food and other substances, prevaccine COVID-19 histories, and past vaccination histories. The second part included questions on local symptoms (pain, swelling, redness, itching, warmth at the injection site, rash, acne, nodule, and abscess), systemic symptoms (fever, nausea, vomiting, headache, myalgia, joint pain, shortness of breath, pale skin, sweaty, cold skin, and swollen lymph nodes), and allergic symptoms (itching, urticaria, angioedema, and maculopapular rash). The exclusion criteria include individuals with low cooperation and orientation, poor communication, vaccination with another brand within 3 months, testing positive for COVID-19 within 3 months, and being under the age of 18. The data were analysed using the SPSS version 25 software. Descriptive statistics were expressed as numbers and percentages. The chi-squared test was used to compare categorical variables. Logistic regression analysis was performed to determine the risk factors. A statistical p value <0.05 was considered significant.

## 3. Results

Of the 403 participants, 134 (33.3%) were female, 269 (66.7%) were male, and the mean age of the participants was 47.7 ± 13.7 (min: 19–max: 85, med: 47; Table 1). 37.9% of the participants had a chronic disease, 8.4% had type 2 diabetes, and 12.7% had hypertension. 26.1% of the participants had a history of chronic medication, 7.4% had a history of allergies, and 27.2% were smokers. A total 84.6% of individuals had received a COVID-19 vaccination and 33.3% had COVID-19 in the past. Of these, 36.5% had received two doses of BioNTech, 31.7% received Sinovac, and 16.4% received both Sinovac and BioNTech vaccines.

Local side effects observed after receiving the Turkovac vaccine were pain at the injection site in 22.8% and swelling and redness in 0.5% of participants. Systemic side effects were weakness or fatigue in 4.2%, muscle or joint pain in 2%, headache in 1.7%, fever in 1%, cough in 0.25%, lymphadenopathy in 0.25%, and urticaria in 0.25% of participants (Table 2). Side effects were observed most frequently within the first 24 h.

In the multinomial logistic regression analysis performed to determine the risk factors, young age (p = 0.02) and allergic diseases (p = 0.024) were predictive of pain at the injection site, type 2 diabetes was predictive of weakness or fatigue (p = 0.017), and female sex was predictive of muscle or joint pain (p = 0.02). Patient occupation, smoking history, and previous vaccinations were not risk factors (p > 0.05).

When the median age and sex of the patients were compared with the side effects, pain at the injection site was significantly more frequent in patients under 47 years of age (p < 0.001). Muscle or joint pain was significantly more common in women (p = 0.011). When local swelling or redness, headache, fever, cough, and lymphadenopathy were compared with the age and sex of the cases, there was no significant difference (p > 0.05; Table 3). When the clinical findings of the cases were compared with the side effects, weakness or fatigue was observed significantly more frequently in those with chronic disease (p = 0.002) and those using regular medication (p = 0.004). Muscle and joint pain were observed significantly more frequently in medicated participants (p = 0.016). No significant correlation was found between pain at the injection site, local swelling or redness, headache, fever, cough, lymphadenopathy and chronic disease, allergy history, medication use, and COVID-19 history (p > 0.05; Table 4).

## 4. Discussion

Previous studies have supported the safety and efficacy of the ERUCoV-Vac vaccine. Powell et al. used BALB/c mice, transgenic mice (K18-hACE2), and ferrets to test the ERUCoV-Vac vaccine and reported that the vaccine was highly immunogenic and had no toxic effects [4]. Özdarendeli et al. reported that administration of two doses of ERUCoV-Vac at 3μg and 6μg 28 days apart, had a good safety profile in a phase 1–2 vaccine study. They also observed over 95% neutralising antibody rates on the 43^rd^ day after the first vaccination [5].

The potential risk of developing side effects from vaccines invokes the antivaccination debate, which is a significant public health problem spanning from the pandemic period. Independent vaccine studies play an important role in eliminating the prejudices of society against vaccine use [6]. To the best of our knowledge, our study is the first independent population-based study to evaluate the side effects of the Turkovac vaccine. Many clinical studies have been shown that inactivated SARS-CoV-2 vaccines have a lower frequency of side effects compared to other types of vaccines [7–9]. Gedik MS et al. reported that of the patients who attended the emergency department after vaccination, 86.9% complained after receiving the Pfizer-BioNTech vaccine (mRNA vaccine) and 13.1% after receiving the Sinovac vaccine [10]. Büyüker SM et al. reported from 602 cases that complaints of physical discomfort and fever were more common in those who received the BioNtech vaccine than those who received the Sinovac vaccine. When all vaccine types were evaluated, the most common side effect was pain at the injection site at 75.19% [11]. Li Q et al. compared the CoronaVac vaccine with the recombinant protein vaccine ZF2001 regarding cutaneous reactions and reported that local reaction was the commonest side effect and more prevalent in the ZF2001 group. However, they also found that morbilliform rash was more common in the CoronoVac group. Most of these reactions were mild and resolved in a short time with conventional treatments [12].

Studies have been conducted to evaluate the local and systemic side effects of inactive COVID-19 vaccines. Batı et al. evaluated the side effects of the inactivated SARS-CoV-2 vaccine Sinovac and reported that the most common local side effect after vaccination was pain (54.6%), whereas the most common systemic side effects were fatigue (39.2%) and headache (34.1%). Also, the frequency of pain was significantly higher in male nurses and those working more than 40 h a week, whereas fever was observed more frequently in nurses with chronic disease [13]. A Turkish study of 780 health-care workers who were administered the CoronaVac vaccine reported the most frequent local side effects to be pain at the injection site (41.5%) while the most frequent systemic side effects were fatigue (23.6%), headache (18.7%), muscle pain (11.2%), and joint pain (5.9%). Local and systemic side effects were observed more frequently in women (67.9%) while younger age and chronic diseases were reported as risk factors [14]. Similarly, CoronaVac vaccinated health-care workers reported the most common side effects as arm pain (55.8%), headache (24.8%), fatigue (18.6%), and joint pain (7.8%) [15]. Others have shown that CoronaVac caused pain at the injection site with a frequency of 23.8% (102/427) after the first dose and 12.8% (52/405) after the second dose. Here the commonest systemic side effect was fatigue with a frequency of 18.2% after the first dose and 10.3% after the second dose [16]. Studies of the Sinaphorm vaccine showed pain at the injection site and fatigue the main side effects, which were more prevalent in women and those younger than 49 years [17]. Furthermore, side effects of the CoronaVac were reported in 24.3% of on Turkish health-care workers and included pain and redness at the injection site, headache, myalgia, joint pain, and palpitation. Females, those under the age of 50, and those with thyroid disorder were at higher risk of suffering side effects [18].

In our study, the total frequency of side effects that were all were mild to moderate, was 32.75% of which 23.3% were local side effects and 9.45% were systemic. Our data suggests that compared with other inactivated vaccines, Turcovac had similar side effects but the risk of serious side effects was low therefore, given the presented data and evidence, the incidence of side effects caused by the Turcovac vaccine is lower compared with mRNA-based vaccines.

The most prevalent local side effects after Turkovac administration were pain and swelling at the injection site, likely related to vaccine reactogenicity. Vaccine reactogenicity represents the inflammatory response to the vaccine. Local inflammation is mediated by macrophages and released cytokines that migrate to the area after vaccination to cause redness and swelling at the injection site [19]. Conversely, injection into a tight muscle can cause more pain than a relaxed muscle and for this reason, it is recommended to lower the arm to reduce pain during injection. Furthermore, storing and injecting vaccines at the optimal temperature can reduce the possibility of pain [20,21].

After vaccination, pyrogenic factors pass into the systemic circulation that affect muscles, vessels, the brain, and other organs causing fever and flu-like symptoms with the release of various inflammatory mediators [19]. The systemic symptoms observed after Turkovac injection include weakness or fatigue, muscle or joint pain, headache, and fever. Furthermore, allergic reactions to vaccines can occur due to microbial antigens, stabilisers, preservatives, adjuvants, and pollutants introduced during vaccine production [22]. In our study, urticarial rash developed in a single case after Turkovac vaccination and was resolved with antihistamines. However, no serious allergic reaction was observed in any of the individuals. We identified the main risk factors for experiencing side effects to be an age younger than 47 years, female sex, chronic disease, and regularly medicated. These risk factors are consistent with published studies and our data also shares a similar profile of the most frequent side effects [14,17,18]. Our data also suggests that local side effects were more common than systemic ones, and that the incidence of serious systemic side effects and the risk of allergy are low. Furthermore, the Turkovac vaccine was well tolerated in individuals over the age of 18. Our data provides solid evidence that can be perceived to change the negative perspective towards vaccines. It would be beneficial to conduct further studies to determine if Turkovac has side effects in other populations.

Our study is limited as the questionnaire only considered adults, the study was single-centred, and the participant number was relatively low. In addition, the evaluation was made after only one dose and the postvaccination follow-up was short. Our study presents valuable data as an independent and community-based study conducted after routine use of the Turcovac vaccine. In addition, although the number of participants is relatively low, our study size is similar to the referenced studies.

## 5. Conclusion

In our study, the most common local side effect of Turcovac vaccination was pain at the injection site, while weakness or fatigue, muscle or joint pain, and headache were the most often systemic side effects. We identified that people under 47 years of age, females, those with chronic diseases, and medicated participants are at a higher risk of experiencing side effects. Our findings reveal that the Turkovac vaccine is well tolerated in the Turkish population and has no serious side effects.

## Figures and Tables

**Table 1:** Demographic and clinical features.

		N	%

**Age, mean + SD (min-max, med)**	47.7 ± 13.7 (19–85, 47)		

**Sex**	Male	134	33.3
	Female	269	66.7

**Occupation**	Health-care worker	10	2.5
	Police	15	3.7
	Teacher	13	3.2
	Officer	51	12.7
	Employee	123	30.5
	Retired	69	17.1
	Not working	122	30.3

**COVID-19 history**	Positive	134	33.3
	Negative	269	66.7

**Allergy history**	Medication allergy	17	4.2
	Food allergy	13	3.2

**Smoking history**	Smoker	110	27.2
	Quit	37	9.2

**Chronic disease**	Diabetes	34	8.4
	Hypertension	51	12.7
	Heart disease	23	5.7
	COPD	27	6.7
	Kidney failure	4	1
	Thyroid disease	7	1.7
	Rheumatic disease	7	1.7

**Medication history**	Antiinflammatory	6	1.5
	Cortisol	10	2.5
	Antihypertensive	50	12.5
	Antihistaminic	5	1.2
	Antidiabetic or insulin	34	8.4

**Previous COVID-19 vaccination history**	BioNTech	147	36.5
	Sinovac	128	31.7
	Sinovac and BioNTech	66	16.4
	None	62	15.4

**Side effect onset time**	<24 h	91	22.6
	1–3 days	35	8.7
	3–7 days	3	0.7
	>7 days	3	0.7

COPD = Chronic obstructive pulmonary disease. N = Number. % = Frequency.

**Table 2:** Frequency of side effects.

	N	%
**Negative**	**271**	**67.25**
**Positive**	**132**	**32.75**
Pain at the injection site	92	22.8
Local swelling, redness	2	0.5
Weakness/fatigue	17	4.2
Muscle/joint pain	8	2
Headache	7	1.7
Fever	4	1
Cough	1	0.25
Lymphadenopathy	1	0.25
Urticaria	1	0.25
TOTAL	**403**	**100**

N = Number. % = Frequency.

**Table 3:** Comparison of side effects according to age and sex.

	<47 years		>47 years			Female		Male		
	N	%	N	%	P	N	%	N	%	P
Pain at the injection site	69	75	23	25	**<0.001**	35	38.4	56	61.6	0.25
Local swelling, redness	1	50	1	50	0.839	0	0	2	100	0.317
Weakness or fatigue	8	47	9	53	0.394	7	41.2	10	58.8	0.478
Muscle or joint pain	6	75	2	25	0.301	6	75	2	25	**0.011**
Headache	3	42.9	4	57.1	0.443	2	28.6	5	71.4	0.791
Fever	2	50	2	50	0.777	1	25	3	75	0.725
Cough	0	0	1	100	0.248	1	100	0	0	0.156
Lymphadenopathy	1	0	0	0	0.385	0	0	1	100	0.480
Urticaria	0	0	1	100	0.24	0	0	1	100	0.48

Chi-Square test.

N = Number. % = Frequency. P = Statistical significance value.

**Table 4:** Comparison of side effects and clinical features.

	Chronic disease			Allergy history			Drug use			History of COVID-19		
	Pos %	Neg %	P	Pos %	Neg %	P	Pos %	Neg %	P	Pos %	Neg %	P
Pain at the injection site	32.6	67.4	0.663	8.7	91.3	0.526	25.9	64.1	0.433	32.6	67.4	0.882
Local swelling, redness	0	100	0.345	50	50	0.139	0	100	1	50	50	1
Weakness/fatigue	64.7	35.3	**0.002**	5.9	94.1	0.830	64.7	35.3	**0.004**	47.1	52.9	0.217
Muscle/joint pain	62.5	37.5	0.05	25	75	0.107	75	25	**0.016**	50	50	0.310
Headache	42.9	57.1	0.485	0	100	0.457	42.9	57.1	0.555	57.1	42.9	0.176
Fever	25	75	1	0	100	1	25	75	1	50	50	0.603
Cough	100	0	0.133	0	100	1	1	0	0.325	0	100	1
Lymphadenopathy	100	0	0.308	0	100	1	100	0	0.325	100	0	0.333
Urticaria	0	100	1	100	0	0.072	0	100	1	0	100	1

Chi-square test.

Pos = ositive. Neg = Negative. % = Frequency . P = Statistical significance value.

## Footnotes

1 Worldometer (2022). Covid-19 Coronavirus pandemic [online]. Website https://www.worldometers.info/coronavirus/#countries . [accessed 03/09/2022].

2 World Health Organization (2022). Coronavirus disease (COVID-19) [online]. Website https://www.who.int/health-topics/coronavirus#tab=tab_2 [accessed

3 Sinovac Research and Development Company Ltd (2022). Safety and Immunogenicity Study of Inactivated Vaccine for Prophylaxis of SARS CoV-2 Infection (COVID-19) [online]. Website https://clinicaltrials.gov/ct2/show/NCT04352608 [accessed 07/06/2022]

4 Türkiye İlaç Ve Tıbbi Cihaz Kurumu (2021). Duyuru [online]. Website https://www.titck.gov.tr/haber/kamuoyunun-dikkatine-22122021142222 . [accessed 01/09/2022].
